# Tracheal Take-Off in an Elderly Patient: A Pig Bronchus or a Separate Right Upper Lobe?

**DOI:** 10.7759/cureus.90400

**Published:** 2025-08-18

**Authors:** Muhammad Mohsin Zahoor, Hira Gul, Brian Casserly

**Affiliations:** 1 Respiratory Medicine, University Hospital Limerick, Limerick, IRL

**Keywords:** accessory right upper lobe, bronchoscopy, congenital airway anomaly, duplicate right upper lobe bronchus, pig bronchus, tracheal bronchus

## Abstract

Tracheobronchial anomalies such as tracheal bronchus, or “pig bronchus,” are extremely rare congenital airway anomalies but are clinically important variants. Distinguishing these from other bronchial variants, such as duplicate or accessory bronchi, can be challenging yet important for diagnosis and clinical management. A pig bronchus is a congenital anomaly in which an aberrant bronchus arises directly from the trachea, most commonly supplying the right upper lobe. Although often asymptomatic, it may predispose individuals to recurrent chest infections due to impaired mucociliary clearance. We report the case of a 76-year-old male with no prior comorbidities who was referred for evaluation of recurrent chest infections requiring multiple courses of antibiotics. Flexible bronchoscopy revealed a right-sided tracheal bronchus arising approximately 2 cm above the carina. A contrast-enhanced CT thorax confirmed the presence of a displaced bronchus supplying the right upper lobe. The patient was managed conservatively with airway clearance strategies and remains under follow-up with reduced infection frequency. This case raises a diagnostic dilemma between a classical pig bronchus and a separate right upper lobe bronchus. While both entities may have similar clinical presentations, differentiating them is important for understanding pathophysiology, planning interventions, and guiding patient management, and should be considered in the differential diagnosis of recurrent infections-even in elderly patients.

## Introduction

A tracheal bronchus is a rare congenital anomaly characterized by an aberrant bronchus arising directly from the trachea, typically located within 2 cm above the carina and most often supplying the right upper lobe. Embryologically, this anomaly is believed to arise from abnormal budding of the tracheobronchial tree between the fourth and sixth weeks of gestation. Normally, the lung buds bifurcate into primary bronchi, but in cases of tracheal bronchus, an accessory or displaced bronchial bud arises prematurely from the trachea. Its estimated prevalence ranges from 0.1% to 2% in the general population [1]. It is often asymptomatic and discovered incidentally; however, it can lead to recurrent infections, atelectasis, or complications during intubation [2]. This case report describes a late-life diagnosis of tracheal bronchus in a patient with recurrent chest infections, emphasizing the importance of recognizing anatomical variants in unexplained respiratory symptoms.

## Case presentation

A 76-year-old male with no known comorbidities was referred by his general practitioner to the respiratory clinic for evaluation of recurrent chest infections over the past one year. He reported multiple episodes of productive cough, fever, and shortness of breath, each requiring antibiotic therapy. These episodes significantly impacted his quality of life. He was a lifelong non-smoker and had no history of chronic lung disease or immunosuppression.

On physical examination, coarse crackles were noted over the right upper chest. Routine blood work was largely unremarkable, except for mild leukocytosis during acute episodes. Chest radiographs showed intermittent right upper lobe infiltrates. Given the recurrent nature of his symptoms, flexible bronchoscopy was performed.

**Figure 1:**
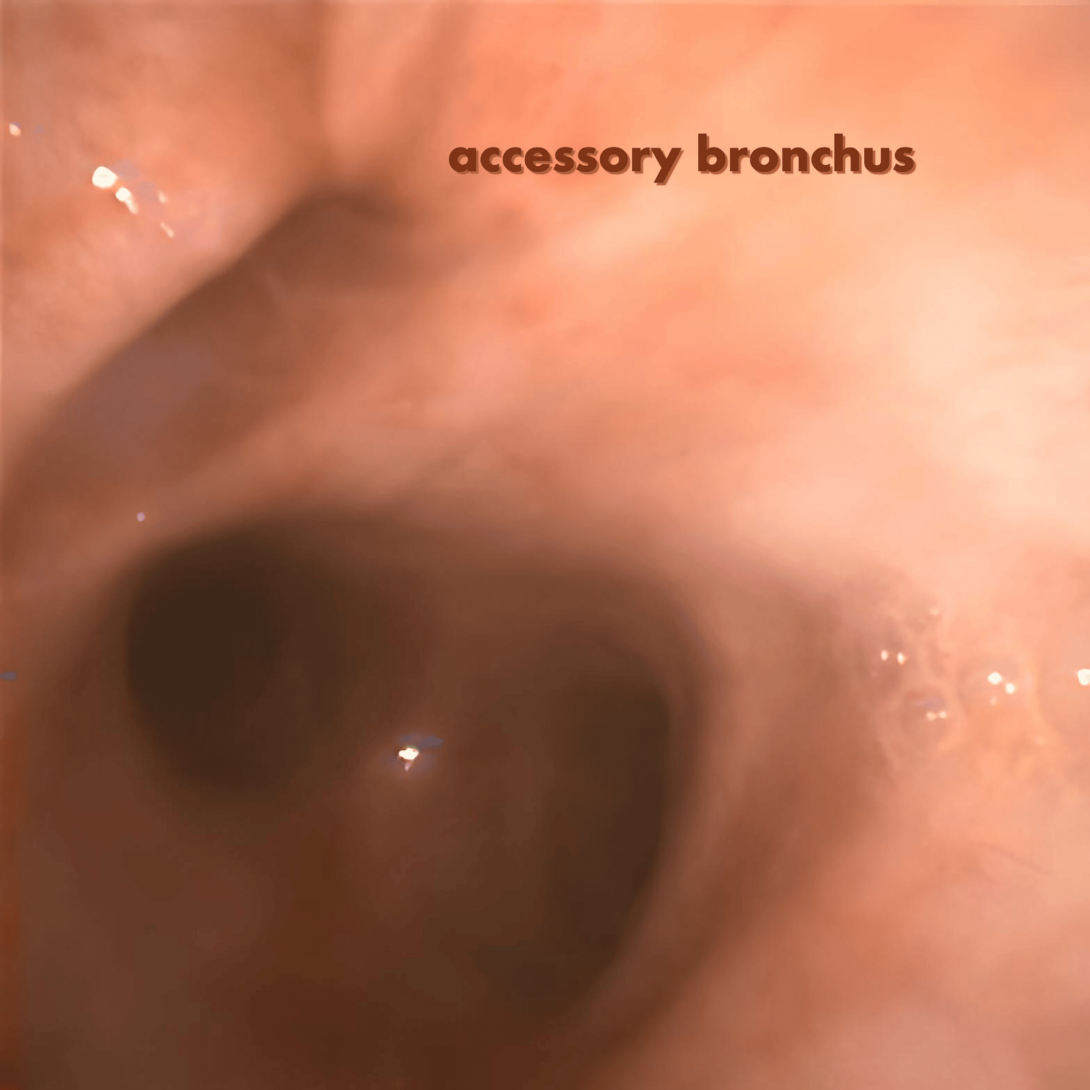
An anomalous (accessory) bronchial opening originating from the right lateral wall of the trachea, approximately 2 cm above the carina.

Bronchoscopy revealed an anomalous bronchial opening arising from the right lateral wall of the trachea, approximately 2 cm above the carina (Figure 1). This bronchus was patent and supplied the right upper lobe. The usual right main bronchus was also present, arising from the carina and bifurcating into segmental bronchi, including a separate branch to the right upper lobe. This dual bronchial supply raised the possibility of a duplicated right upper lobe bronchial system.

**Figure 2:**
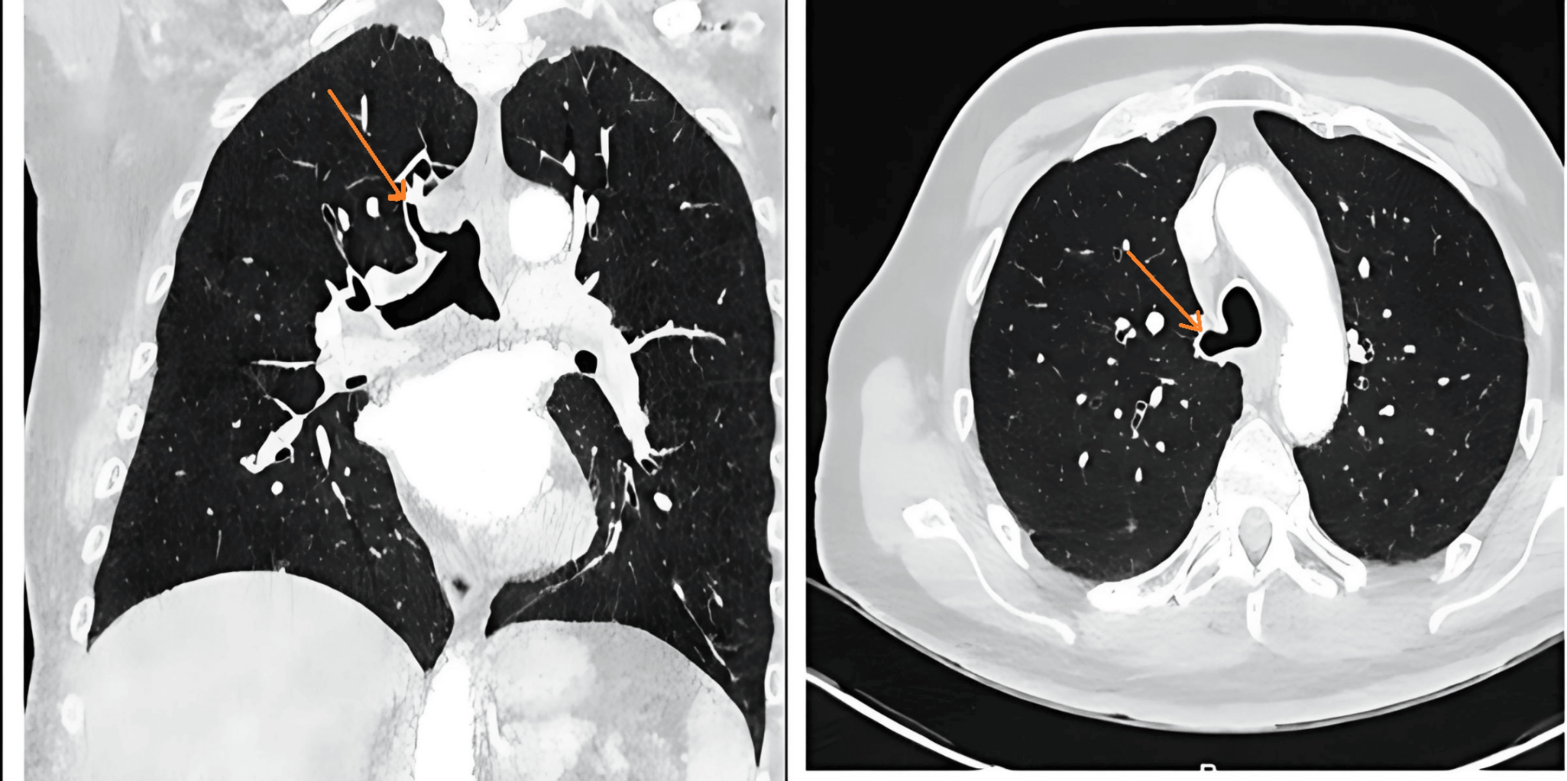
CT of the thorax confirmed a displaced tracheal bronchus, commonly referred to as a pig bronchus, supplying the right upper lobe.

A contrast-enhanced CT thorax confirmed the presence of a displaced tracheal bronchus - commonly known as a *pig bronchus *- supplying the right upper lobe (Figure 2). However, the presence of two separate bronchial supplies to right upper lobe arises the question: is this a classic pig bronchus, or could it represent a true anatomical variant with a duplicated right upper lobe bronchial system. No other structural abnormalities or bronchiectasis were noted.

**Figure 3:**
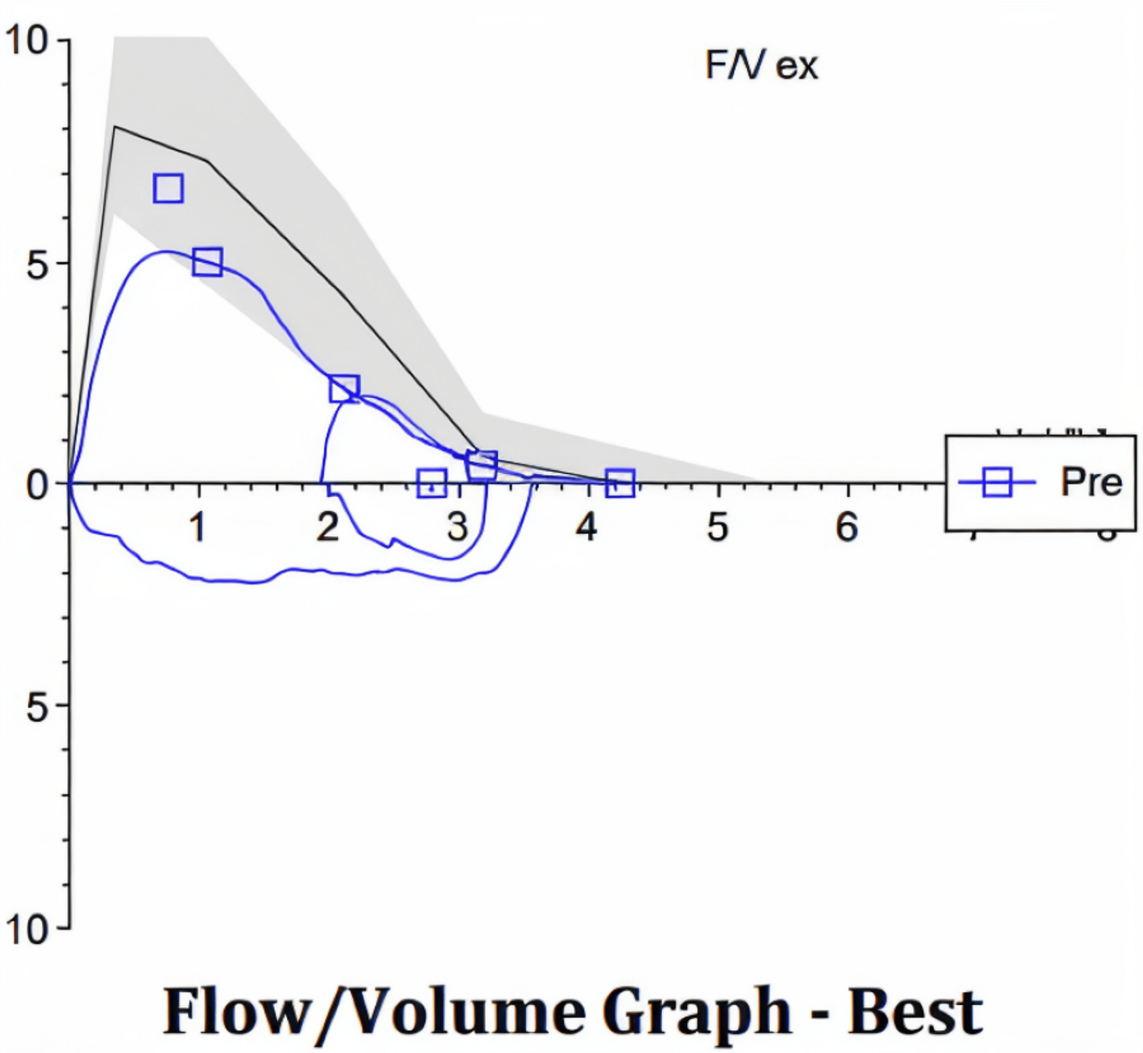
Abnormal flow-volume loop on spirometry, supporting possible airway obstruction.

Pulmonary function testing later revealed abnormal flow-volume loop in spirometry, supporting possible airway obstruction (Figure 3).

The patient was managed conservatively with airway clearance techniques, including chest physiotherapy and a mucolytic agent. He was educated about the nature of the anomaly and remains under follow-up, with a significant reduction in the frequency of infections.

## Discussion

Tracheal bronchial anomalies are rare variants of the tracheobronchial tree, often discovered incidentally during radiological or bronchoscopic evaluation for other respiratory symptoms. Tracheal bronchus, also known as pig bronchus or bronchus suis, was first described by Sandifort in 1785 and may be classified as either supernumerary (accessory bronchus) or displaced (replacing the normal upper lobe bronchus) [1]. The displaced type, often referred to as pig bronchus, is more clinically significant as it supplies the entire right upper lobe and may impair mucociliary clearance.

In this case, a bronchial branch originating approximately 2 cm above the carina and supplying the right upper lobe raises a diagnostic consideration between a classic pig bronchus and a potentially duplicate right upper lobe bronchus. What also makes this case unique and diagnostically intriguing is the presence of an additional anatomically normal right upper lobe bronchial supply arising from the right main bronchus, suggesting dual segmental supply.

The distinction between a pig bronchus and a duplicate right upper lobe bronchus is more than academic. While both can lead to similar clinical presentations, they may have different embryological origins and implications for airway management, surgical planning, and interpretation of imaging studies [1,2]. 

The presence of a duplicated bronchial pathway to the right upper lobe has important clinical implications, especially in elderly patients who often present with multiple comorbidities. From an airway management perspective, such an anomaly may complicate endotracheal intubation or bronchoscopy, as standard anatomical landmarks may not reliably guide tube placement or suctioning. Misidentification of the anomalous bronchus could lead to incomplete ventilation or aspiration risk if secretions accumulate in an unrecognized segment.

Furthermore, in patients undergoing pulmonary interventions, such as lobectomy, segmentectomy, or bronchial stenting, precise anatomical mapping becomes critical to avoid inadvertent damage to functional lung tissue. In elderly individuals with reduced pulmonary reserve or chronic respiratory conditions (e.g., COPD), preserving optimal ventilation and minimizing procedural risks are paramount. Awareness of such variations can inform preoperative planning, guide imaging interpretation, and improve outcomes in both emergent and elective settings.

A true pig bronchus is mainly a supernumerary bronchus without segmental distribution, whereas a separate bronchial system suggests bifurcation of developmental anatomy, leading to partial lobar duplication.

CT imaging, especially with coronal reconstructions, is the gold standard for diagnosis. Bronchoscopy provides direct visualization and helps rule out other airway abnormalities. Advanced techniques like 3D reconstruction from high-resolution computed tomography (HRCT) or bronchoscopy can assist in clarifying the branching pattern, but sometimes the distinction remains subtle [3].

Management of such cases depends on symptom severity. Asymptomatic patients often require observation, whereas symptomatic patients require airway clearance, antibiotics, and surgical resection (in rare cases) [4].

In this case, the patient’s recurrent infections were likely due to impaired drainage of the right upper lobe via the anomalous bronchus. Abnormal flow-volume loop in spirometry, supporting possible airway obstruction due to secretions. Conservative management, i.e., postural drainage and incentive spirometry, proved effective.

## Conclusions

This case highlights the presence of unusual bronchial anatomy with a diagnostic ambiguity between a tracheal (pig) bronchus and a duplicate/separate right upper lobe bronchial supply. Both are exceedingly rare and may predispose to recurrent respiratory infections. The importance of considering congenital airway anomalies such as these in elderly patients with unexplained recurrent respiratory infections is essential not only for diagnostic clarity but also for tailoring patient management. Awareness of these anomalies is also critical for anesthesiologists during airway management and is equally important for cardiothoracic surgeons when performing lung resection surgery. Further studies may aid in classifying such anatomical variants.
